# Myosin-Vb functions as a dynamic tether for peripheral endocytic compartments during transferrin trafficking

**DOI:** 10.1186/1471-2121-9-44

**Published:** 2008-08-07

**Authors:** D William Provance, Erin J Addison, Patrick R Wood, David Z Chen, Colleen M Silan, John A Mercer

**Affiliations:** 1 McLaughlin Research Institute, Great Falls, MT, USA; 2 University of Washington School of Medicine, Seattle, WA, USA; 3 Amherst College, Amherst, MA, USA

## Abstract

**Background:**

Myosin-Vb has been shown to be involved in the recycling of diverse proteins in multiple cell types. Studies on transferrin trafficking in HeLa cells using a dominant-negative myosin-Vb tail fragment suggested that myosin-Vb was required for recycling from perinuclear compartments to the plasma membrane. However, chemical-genetic, dominant-negative experiments, in which myosin-Vb was specifically induced to bind to actin, suggested that the initial hypothesis was incorrect both in its site and mode of myosin-Vb action. Instead, the chemical-genetic data suggested that myosin-Vb functions in the actin-rich periphery as a dynamic tether on peripheral endosomes, retarding transferrin transport to perinuclear compartments.

**Results:**

In this study, we employed both approaches, with the addition of overexpression of full-length wild-type myosin-Vb and switching the order of myosin-Vb inhibition and transferrin loading, to distinguish between these hypotheses. Overexpression of full-length myosin-Vb produced large peripheral endosomes. Chemical-genetic inhibition of myosin-Vb after loading with transferrin did not prevent movement of transferrin from perinuclear compartments; however, virtually all myosin-Vb-decorated particles, including those moving on microtubules, were halted by the inhibition. Overexpression of the myosin-Vb tail caused a less-peripheral distribution of early endosome antigen-1 (EEA1).

**Conclusion:**

All results favored the peripheral dynamic tethering hypothesis.

## Background

Molecular motors generally are thought to be recruited to vesicles or organelles to provide directional movement; however, this perspective is complicated by evidence showing that multiple motors, using multiple cytoskeletal substrates, are found on individual vesicles and organelles [1-7]. While kinesins and dyneins clearly transport cargo for long distances *in vivo*, there is surprisingly little evidence for such a role for unconventional myosins in higher eukaryotes [8]. Biophysical studies have shown that many myosin head domains bind more tightly to actin in response to loading [9-12], but these adaptations usually are interpreted as promoting processive transport of cargo over long distances. However, the biophysical data also are consistent with adaptation to function as dynamic tethers or tensioners between actin filaments and other cytoplasmic structures. In this context, tethering is distinct from docking, in that it may simply represent a net balance of forces, movements, and/or positions. Accordingly, retention (and active transport) within cortical actin might prevent endosomes from encountering microtubules, so tethering may represent an effect rather than a distinct molecular mechanism. Actual point-to-point transport of cargo by unconventional myosins in a cellular context might be relatively rare; for example, to reposition the myosin in the absence or reduction of load, halting as a new load is sensed.

Myosin-Vb, originally named myr 6 [13], is a member of one of the most ancient divisions of the myosin superfamily [14], with diverse cellular functions. It interacts with the brain-expressed RING finger protein BERP, Rab11a, Rab11a-FIP2, Rab11b, Rab25, and Rab8a [15-19]. It has been implicated in recycling of transferrin and its receptor [16,18,20], the chemokine receptor CXC2 [21], HIV Vpu [22], acetylcholine receptors [23], the polymeric IgA receptor [16,24], and the alpha-amino-3-hydroxy-5-methylisoxazole-4-propionic acid (AMPA)-type glutamate receptor subunit GluR1 [25]. It also has been implicated in formation of bile canaliculi [26].

Overexpression of tail fragments of unconventional myosins has been the standard technique for their inhibition, and data from these experiments are usually interpreted in the context of point-to-point transport. For myosin-Vb in transferrin trafficking, overexpression of a tail fragment in HeLa cells caused accumulation of transferrin in perinuclear compartments, suggesting that myosin-Vb functions in the transport of vesicles between perinuclear recycling endosomes and the plasma membrane [16]. By contrast, we adapted a chemical-genetic method pioneered by Shokat and colleagues for kinases [27,28] to unconventional myosins, allowing us to acutely and specifically induce tight binding of a sensitized mutant myosin to actin by microinjection or dialysis of an ADP analog [29-32]. When we inhibited the sensitized mutant myosin-Vb (also dominant-negative inhibition), it prevented accumulation of transferrin-positive vesicles and organelles in the perinuclear region [20]. This result was inconsistent with the transport hypothesis, because if myosin-Vb is required for transport between perinuclear compartments and the plasma membrane, induction of tight binding to actin should have caused transferrin to accumulate in perinuclear compartments.

These apparently contradictory results could be reconciled if myosin-Vb acts peripherally as a dynamic tether that antagonizes the retrograde transport of transferrin to perinuclear compartments, possibly by holding the parental organelle in the periphery during fission. We also observed an increase in plasma-membrane transferrin receptor upon myosin-Vb inhibition [20], suggesting that chemical-genetic inhibition had shunted trafficking to the rapid peripheral pathway [33]. The tail-fragment overexpression data can be explained as release of the peripheral endocytic compartments from actin, allowing entire peripheral endosomes to be transported to the perinuclear region.

**Figure 1:**
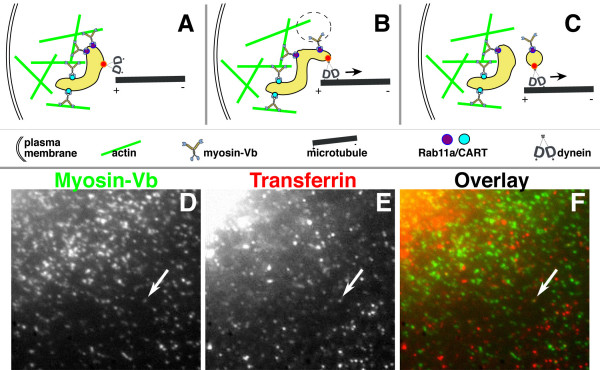
**Model and normal distributions of myosin-Vb and transferrin**. **(A) **Peripheral endosomes are retained in the periphery by multiple myosin-Vb motors whose heads periodically detach from actin as they go through the ATPase cycle, but usually reattaching. **(B) **Dynein (or a minus-end-directed kinesin) attaches to a microtubule and exerts retrograde force. Occasionally, adjacent myosin-Vb detaches from actin (dotted circle), allowing dynein to pull it away from the actin filament. **(C) **Following fission, the daughter vesicle moves retrogradely, carrying myosin-Vb as a passenger. **(D, E, F) **In HeLa cells expressing low levels of eGFP-myosin-Vb, colocalization between myosin-Vb (green) and transferrin (red) is rare (arrow) and transient (also see Additional file 1).

Our hypothesis is illustrated in Fig. 1. Peripheral endosomes are retained in the periphery by multiple myosin-Vb motors whose heads periodically detach from actin (green) as they go through the ATPase cycle, but usually rapidly reattach, as suggested by biophysical data (Fig. 1A). In Fig. 1B, dynein (or a minus-end-directed kinesin; its head domain is shown as the letter "D") attaches to a microtubule and exerts retrograde force. Occasionally, adjacent myosin-Vb detaches from actin (dotted circle) and cannot reattach because dynein has pulled it away from the actin filament. The remaining myosins hold the bulk of the endosome in place. In Fig. 1C, fission has occurred, and the daughter vesicle moves retrogradely; after the switch to microtubules, myosin-Vb is carried along as a passenger. Although we hypothesize that myosin-Vb primarily functions as a dynamic tether, our model does not preclude myosin-Vb-dependent meandering within the peripheral actin network. We have diagrammed two different means of binding myosin-Vb to the endosome as cyan and purple circles, since two different means have been demonstrated experimentally: Rab11a [16] and the CART complex [18].

In this study, we have employed three different perturbations of myosin-Vb function to further test the dynamic tethering hypothesis, which makes clear predictions: first, overexpression of full-length, functional myosin-Vb will prevent transferrin from reaching perinuclear compartments; second, chemical-genetic inhibition of sensitized mutant myosin-Vb in cells **after **transferrin loading (in our previous study, it was done **before **transferrin loading) will neither cause accumulation of transferrin in perinuclear compartments nor prevent transferrin from moving from perinuclear compartments to the plasma membrane; and third, overexpression of the myosin-Vb tail fragment will cause at least some peripheral endocytic markers to assume more perinuclear distributions. The new data generally contradict the transport hypothesis. In addition, our data suggest that members of the myosin-V family may play a ubiquitous function in modulating vesicle transport along microtubules, as they are available to interact with passing actin filaments as passengers. Applied more broadly, our data suggest that identifying endocytic compartments by their positions within the cytoplasm may be unreliable in the context of significant experimental disruptions.

## Results and Discussion

We have shown that expression of low levels of exogenous myosin-Vb (25–40% of endogenous levels) does not alter the trafficking of transferrin [20]. However, the dynamic tethering hypothesis predicts that exceeding endogenous levels with wild-type exogenous myosin-Vb will alter the balance of forces, reducing the extent and/or rate of retrograde movement from peripheral to perinuclear compartments. To test this prediction, we increased the amount of myosin-Vb associated with those compartments by transiently transfecting HeLa cells with a full-length, wild-type myosin-Vb construct. To allow imaging of live cells, we used a construct with an N-terminal eGFP tag [16]. We compared the distribution of eGFP-tagged myosin-Vb with that of our C-terminal -tagged (V5 and 6x-His) version [20], and observed no significant differences (data not shown). At low levels of eGFP-myosin-Vb expression, we observed only occasional, highly dynamic, colocalization of myosin-Vb and transferrin (arrows, Fig. 1D,E,F; Additional file 1).

**Figure 2:**
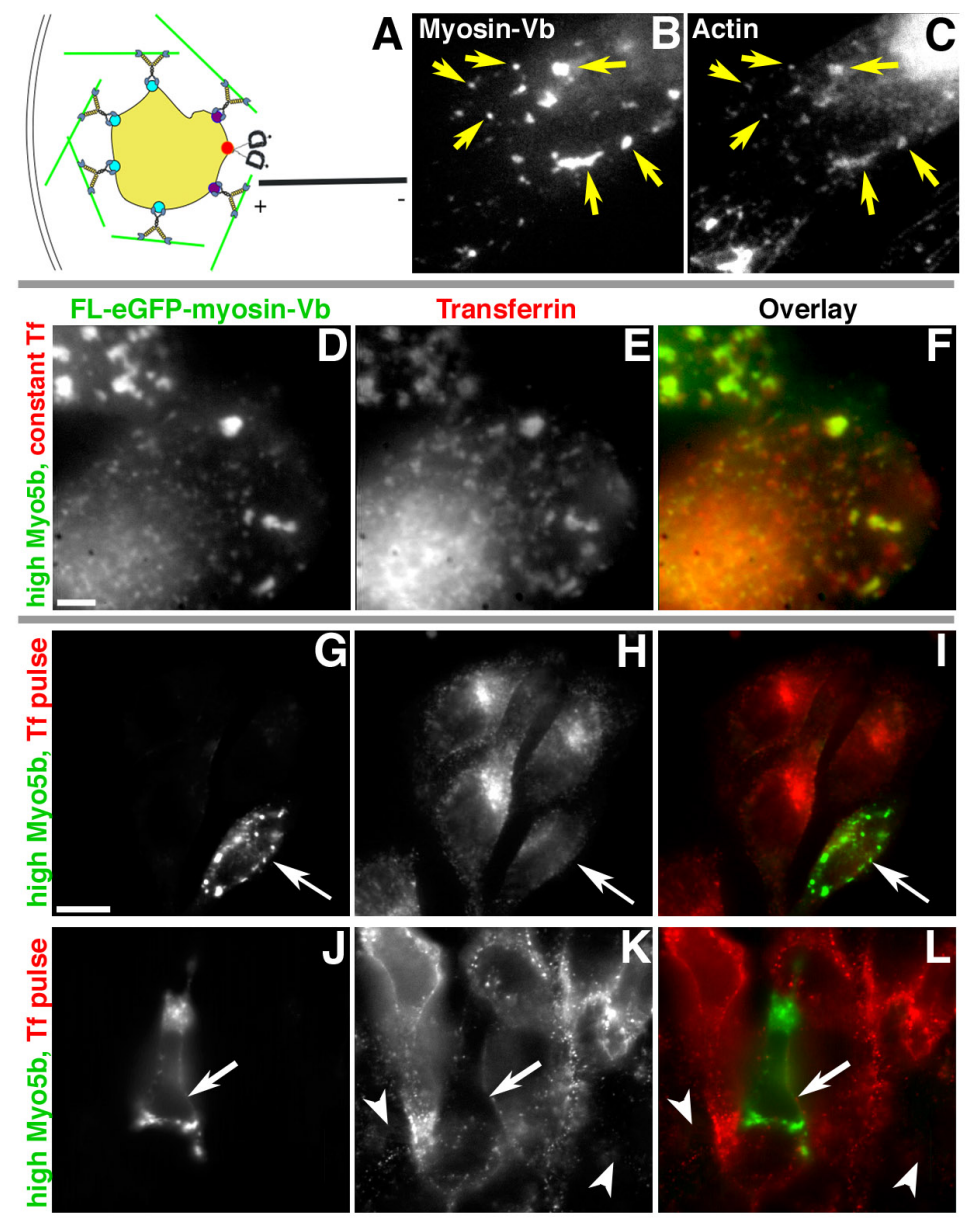
**Overexpression of full-length, wild-type eGFP-myosin-Vb causes coalescence of peripheral endocytic compartments and inhibits perinuclear accumulation of transferrin**. HeLa cells were transiently transfected with full-length, wild-type myosin-Vb tagged with eGFP and imaged 24 h after transfection. **(A) **Diagram depicting predicted results. **(B, C) **Colocalization of actin on enlarged compartments (arrows) with eGFP-myosin-Vb. **(D, E, F) **In cells expressing high levels of eGFP-myosin-Vb coincident with exposure to transferrin (arrows), large, peripheral organelles decorated with myosin-Vb also contain transferrin. **(G, H, I, J, K, L) **In cells exposed to a 1-min pulse of transferrin 24 h after transfection and 10 min before imaging, transfected cells (arrows) contain large, peripheral organelles decorated with myosin-Vb that lack transferrin. Cells expressing lower levels of myosin-Vb (arrowheads, panel K; too low to be seen in panel J) accumulate less transferrin than the surrounding untransfected cells (arrowheads). Bar, 15 μm.

Figure 2 and Additional files 2, 3, 4, 5, 6, 7, 8 show transferrin accumulation in peripheral compartments as a function of the overexpression level of eGFP-myosin-Vb, which the dynamic tethering hypothesis predicts will cause the coalescence and caging of peripheral endosomes by actin (Fig. 2A). A coalescence of actin around the enlarged peripheral endosomes is shown by the colocalization of myosin-Vb and actin (Fig. 2B,C).

HeLa cells that endocytosed fluorescent transferrin **before and during **overexpression of eGFP-myosin-Vb sequestered transferrin in large peripheral compartments decorated with myosin-Vb (Fig. 2D,E,F; Additional file 2), suggesting that fission of the compartments in which myosin-Vb and transferrin normally transiently colocalize (arrow, Fig. 1D,E,F; Additional file 1) was inhibited. By contrast, when transferrin was introduced **after **overexpression of myosin-Vb, transferrin was not colocalized with myosin-Vb in the enlarged peripheral compartments (arrows, Fig. 2F–K; Additional files 3, 4, 5). In addition, transferrin failed to accumulate in perinuclear compartments. As a negative control, we expressed a truncated myosin-Vb consisting of the head domain and first IQ domain, which had no effect on transferrin localization (data not shown). These data suggest that that overexpression of myosin-Vb prevents transferrin from both entering into and exiting from a normally dynamic, short-lived endocytic compartment.

In isolation, the static images shown in Figure 2 can be fit to the anterograde transport model if overexpression caused rapid transport of transferrin from perinuclear compartments while delaying its passage through cortical actin. However, Additional files 3, 4, 5 show that transferrin is not reaching perinuclear compartments.

As these data suggest that fission of vesicles from peripheral endocytic compartments and/or their transport to perinuclear compartments had been prevented by increased tethering to cortical actin, we examined the distribution of the endocytic markers Rab11a, Rab4, and Rab5. Cotransfections with eGFP-myosin-Vb and the recycling endosome marker mRFP-Rab11a showed virtually complete colocalization at high levels of myosin-Vb expression (Additional file 6). By contrast, little colocalization was observed in cells cotransfected with eGFP-myosin-Vb and the early endosome markers mRFP-Rab4 (Additional file 7) and mRFP-Rab5 (Additional file 8), suggesting that trafficking through early endosomes was not prevented. The videos also show that the enlarged endosomes are relatively static, consistent with increased tethering forces and caging by actin.

**Figure 3:**
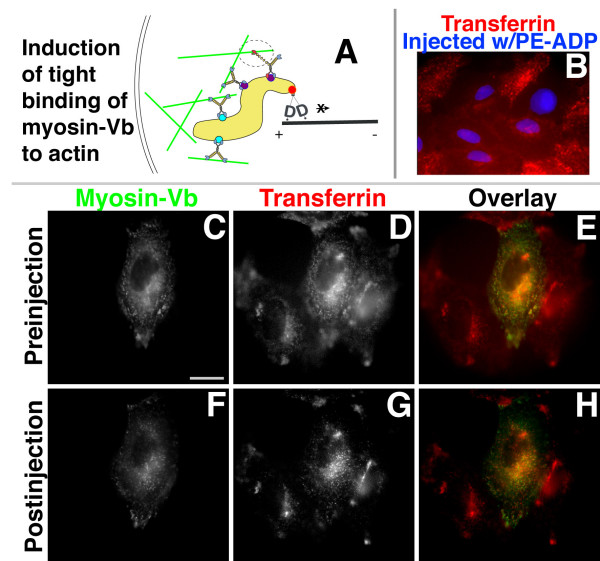
**Inhibition of myosin-Vb after loading with transferrin does not prevent transit from perinuclear recycling endosomes**. HeLa cells transiently expressing sensitized myosin-Vb were loaded with Alexa 546-transferrin, washed, incubated in growth medium for 30 min, and imaged for myosin-Vb and transferrin. **(A) **Diagram depicting predicted results; the sensitized mutant myosin-Vb is shown in red and PE-ADP is shown as a green circle. **(B) **Inhibition of accumulation of transferrin (red) added after myosin-Vb inhibition by microinjection of PE-ADP^2^. Injected cells have blue nuclei. **(C, D, E, F, G, H) **The cell expressing sensitized myosin-Vb (center, panel C) was immediately injected with PE-ADP and the same field was imaged 30 min later (F, G, H). Panels C and D are overlaid in panel E, and panels F and G are overlaid in panel H. Bar, 15 μm.

In a previous study, we used a chemical-genetic approach to show that induction of tight binding of sensitized myosin-Vb to actin, **before **addition of transferrin, prevented transferrin from accumulating in perinuclear compartments [20]. Our hypothesis is diagrammed in Fig. 3A, and the effect of inhibition before transferrin uptake, demonstrated previously, is shown in Fig. 3B. If myosin-Vb is required for transport from perinuclear compartments to the plasma membrane, then inducing tight binding of myosin-Vb to actin **after **transferrin loading should increase transferrin accumulation in perinuclear compartments, just as myosin-Vb tail overexpression does. We therefore transfected HeLa cells with Y119G sensitized mutant (Fig. 3) and wild-type control (not shown) myosin-Vb, loaded them with fluorescent transferrin, and microinjected the specific inhibitor of Y119G myosin-Vb, ***N***^6^-(2-phenylethyl)-ADP (PE-ADP) [20]. Only cells with a punctate eGFP localization, representing lower expression levels, were chosen for microinjection. When PE-ADP was injected 10 min (data not shown) and 30 min (Fig. 3D,E,F) following the addition of transferrin, we still observed a decrease in fluorescence intensity in the perinuclear region of the transfected and injected cells (Fig. 3D,E,F) as well as rapid movement of transferrin when it did not colocalize with myosin-Vb (Additional file 9). These data, as well as the limited colocalization between transferrin and myosin-Vb, indicate that myosin-Vb activity is not required to transport transferrin from perinuclear compartments to the plasma membrane. These data are much more consistent with the peripheral tethering hypothesis, because the peripheral site of myosin-Vb function has been bypassed by loading with transferrin before induction of tight binding of myosin-Vb to actin.

**Figure 4:**
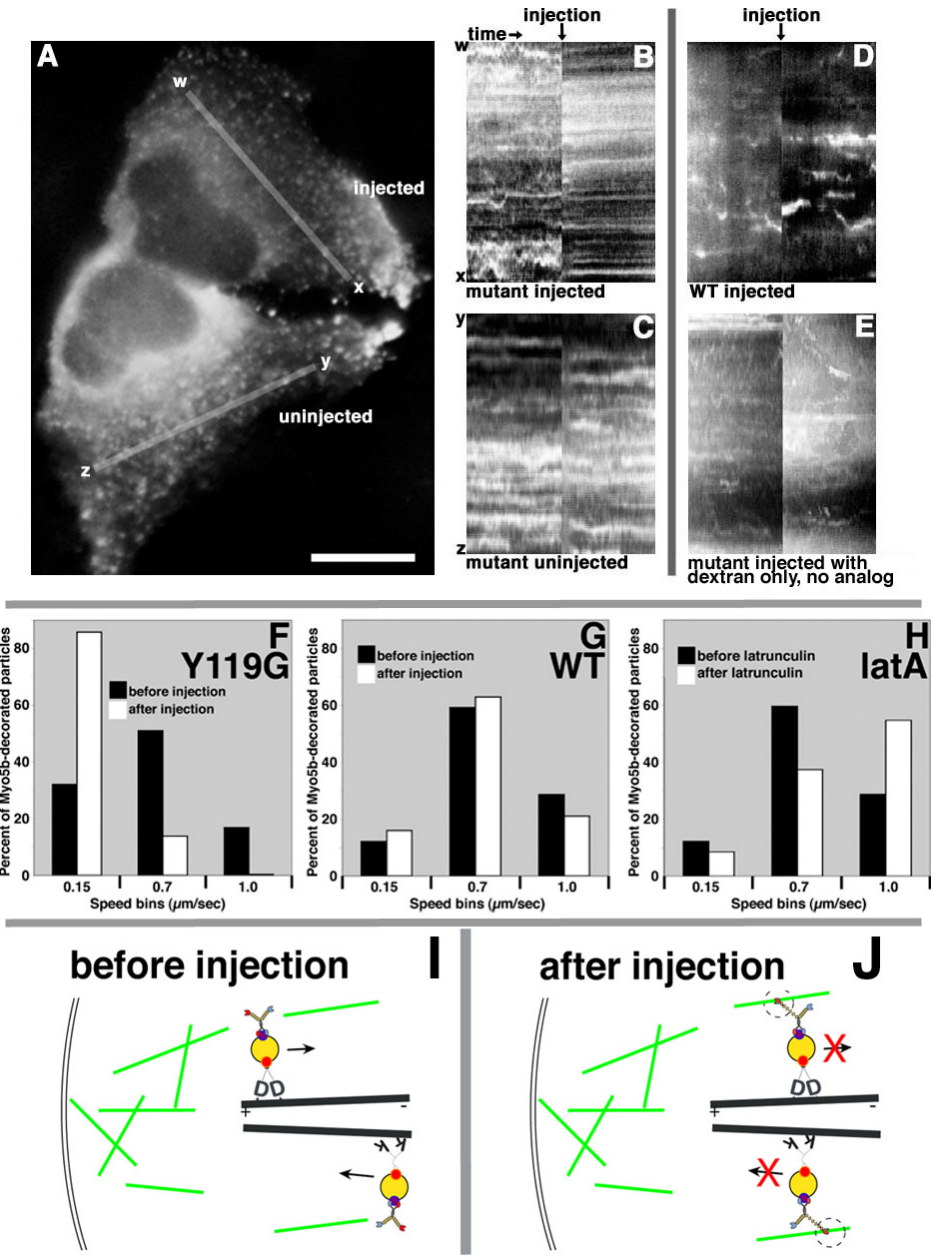
**Chemical-genetic inhibition of myosin-Vb halts myosin-Vb-decorated particles, including those being transported via microtubules**. **(A) **Representative image of two HeLa cells expressing sensitized Y119G mutant eGFP-myosin-Vb before injection of the upper cell with PE-ADP. Bar, 15 μm. **(B and C) **Kymographs from the cells shown in panel A (y axes represent lines wx and yz from panel A). **(D and E) **Kymographs from additional negative control cells expressing wild-type myosin-Vb injected with PE-ADP and wild-type myosin-Vb injected with dextran respectively. **(F and G) **Histograms of instantaneous speeds of myosin-Vb-labeled vesicles before (black bars) and after (white bars) PE-ADP injection in cells expressing wild-type and Y119G mutant eGFP-myosin-Vb respectively; Speeds were measured for 1178 (before injection) and 621 particles (after) for panel F, and 717 and 551 respectively for panel G. **(H) **Instantaneous speeds of wild-type eGFP-myosin-Vb-labeled vesicles before (black bars) and after (white bars) depolymerization of actin by latrunculin A; the Y119G mutant gave indistinguishable results (data not shown). Speeds were measured for 707 (before) and 206 (after) particles. **(I and J) **Diagrams depicting additions to the dynamic tethering hypothesis to accommodate these data. Kinesin is represented with the letter "k" for the head domain.

While the inhibition of the Y119G sensitized mutant myosin-Vb in preloaded cells did not cause transferrin accumulation in perinuclear compartments, the data were not as simple as they were predicted to be by the dynamic tethering hypothesis, as myosin-Vb inhibition retarded the depletion of transferrin from perinuclear compartments relative to control cells (Fig. 3G,H). Upon closer examination, our induction of binding of myosin-Vb to actin had the general effect of halting nearly all motion of myosin-Vb-decorated structures within the cell (Fig. 4A). The motility of eGFP-myosin-Vb before and after microinjection was analyzed using kymographs (Fig. 4B,C for the cells shown in Fig. 4A, Fig. 4D,E for additional negative control cells; also see Additional files 10, 11, 12). Binned measurements of instantaneous particle speeds in the presence and absence of PE-ADP (Fig. 4F, Additional files 10 and 11) show that not only was slower actin-based motility (0.15 – 0.3 μm/s) inhibited, but higher-speed movements of myosin-Vb-decorated particles caused by microtubule-based motors (> 0.7 μm/s) were halted as well. No such inhibition was observed under control conditions, which included cells expressing Y119G myosin-Vb after injection of vehicle plus fluorescent Dextran without PE-ADP (data not shown), as well as cells expressing wild-type myosin-Vb after PE-ADP injection (Fig. 4G, Additional file 12).

The arrest of microtubule-based motility of myosin-Vb-decorated particles was unexpected, and we initially suspected that it might have been an artifact of high effective ADP concentration in the form of the microinjected PE-ADP analog. To test the hypothesis that myosin-Vb interacts transiently with actin filaments during microtubule-based transport under normal conditions, we measured the speeds of particles decorated with wild-type eGFP-tagged myosin-Vb before and after the addition of latrunculin A. If myosin-Vb (or other myosins) normally interacts with actin filaments, latrunculin A treatment should increase both mean speed and the proportion of vesicles moving at 0.7–1.0 μm/sec. This prediction was confirmed, as latrunculin treatment nearly doubled the proportion of particles exhibiting rapid movement (Figure 4H), in contrast with results from melanosome transport in fish melanophores [34]. The modification of the dynamic tethering hypothesis to account for these data is diagrammed in Fig. 4I and 4J.

**Figure 5:**
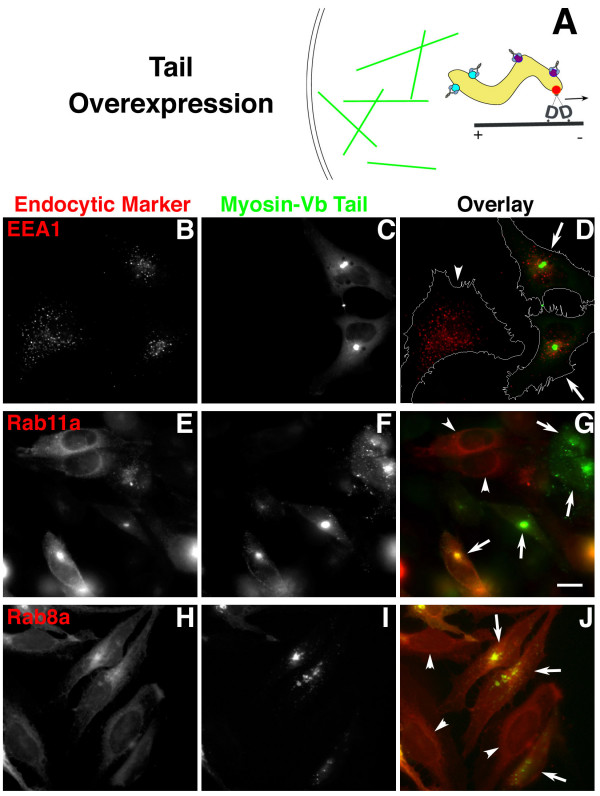
**eGFP-myosin-Vb tail overexpression displaces EEA1 and Rab11a to more perinuclear positions**. HeLa cells were transfected with the eGFP-myosin-Vb tail construct and allowed to express overnight. **(A) **Diagram depicting displacement of peripheral endosomes. **(B) **Immunofluorescent detection of EEA1. (**E, F, G) **Cotransfection with the mRFP-Rab11a construct. **(H, I, J) **Cells cotransfected with the mRFP-Rab8a construct. **(C, F, I) **eGFP-myosin-Vb tail. **(D, G, J) **overlays of B+C, E+F, and H+I respectively; arrows, cells expressing the myosin-Vb tail fragment; arrowheads, control cells not expressing the tail. Bar, 15 μm.

The dynamic tethering hypothesis further predicts that some markers found in peripheral endocytic compartments are likely to be shifted to a more perinuclear distribution by myosin-Vb tail overexpression (Fig. 5A). We tested this prediction for early endosomal antigen-1 (EEA1), which had a dispersed pattern in control cells (Fig. 5B,C,D, arrowhead), while in cells expressing the eGFP/myosin-Vb tail chimera [16], EEA1 was much more concentrated, in an asymmetric pattern primarily on one side of the nucleus (Fig. 5B,C,D, arrows).

Based on the change in distribution of EEA1 coupled with its failure to colocalize with the myosin-Vb tail, we hypothesize that in the presence of the tail, endosomes still are transported to more perinuclear regions of the cytoplasm, but the fission between their domains that normally occurs in peripheral regions occurs in a more perinuclear location. We then confirmed the effect of the myosin-Vb tail on Rab11a redistribution. As observed by Lapierre et al., the dispersed pattern observed in untransfected control cells (Fig. 5E,F,G, arrowheads) was changed to a more perinuclear pattern by overexpression of the eGFP/myosin-Vb tail (arrows).

We next examined Rab8a, which has been shown to interact *in vitro *with myosin-Vb [19]. We observed a nearly normal distribution of Rab8a despite the overexpression of the myosin-Vb tail (Fig. 5H,I,J). These results are consistent with the differences between Rab11a and Rab8a compartments and pathways observed by Roland et al., and indicate that the affinity of the myosin-Vb tail domain for Rab8a is much lower than its affinity for Rab11a. This result also is consistent with their inability to observe interaction between myosin-Vb and Rab8a in a cellular context.

**Figure 6:**
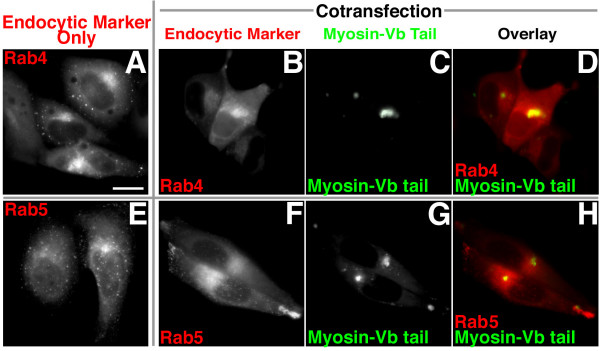
**(A-H) Overexpression of eGFP-myosin-Vb tail causes a slight shift in Rab4 distribution, but has little effect on Rab5 distribution**. HeLa cells were transiently transfected with eGFP-myosin-Vb tail fragment **(B,C,D,F,G,H) **and mRFP-Rab4 **(A,B,C,D) **or mRFP-Rab5 **(E,F,G,H) **and imaged after overnight incubation. Bar, 15 μm.

Since mosaic endosomes have been observed with every possible combination of Rab4, Rab5, and Rab11a [35], we examined Rab4 and Rab5 distribution. Overexpression of the myosin-Vb tail produced a slight alteration in the distribution of Rab4 (Fig. 6A,B,C,D), but no significant effect on Rab5 distribution (Fig. 6F,G,H), which is puzzling given the association between EEA1 and Rab5 [36].

To summarize our model, myosin-Vb is associated with multiple compartments, of which only some are involved in transferrin trafficking. Myosin-Vb primarily tethers a subset of peripheral, Rab11a-positive endocytic compartments to cortical actin, opposing forces from dynein or minus-end-directed kinesins and retaining the compartment in the actin-rich periphery. This is analogous to the mechanism of Velcro™, except that instead of hooks bending, the myosin-Vb heads are going through the ATPase cycle and periodically releasing from actin. In this analogy, overexpression of full-length, wild-type myosin-Vb (Fig. 2) causes greater retention of normally endocytic compartments in the periphery, leading to their coalescence, because the increase in number of myosins outweighs their individual cycling off and back onto actin. These data strongly suggest that while this compartment is normally rare and transient (Fig. 1), all transferrin still must pass through it to reach perinuclear compartments. Both our overexpression of myosin-Vb and overexpression of the myosin-Vb tail create artifacts. In the former case (Fig. 2), caging by actin causes coalescence and blockage of both entry and exit; while in the latter case [16], release from actin causes what we believe to be virtually the same compartment to collapse to a perinuclear location. Chemical-genetic inhibition is analogous to preventing individual Velcro™ hooks from bending, but does not prevent entry and exit into this compartment via the peripheral pathway [20].

Myosin-Va, the founding member of this myosin family, appears to have a similar function, albeit involving different compartments. In melanocytes, we first suggested a peripheral tethering function for myosin-Va based on the mutant phenotype, a perinuclear accumulation of melanosomes [37]. The best-characterized system for melanosome transport has been Xenopus melanophores [4,5,38,39], with similar, but less dynamic, results from murine melanocytes [40]. In both cases, myosin-Va function was hypothesized to provide not only peripheral capture, but transport within the periphery as well. While pauses in microtubule-based movement attributed to myosin-Va have been observed [41,42], these studies represent the first such observation for myosin-Vb. In general, myosin-Vb appears to perform the same function in the endocytic pathway as myosin-Va performs in exocytic pathways, and our future experiments will test the validity of our generalization. In a technical context, our results suggest that membrane compartments cannot necessarily be reliably identified by their locations within the cytoplasm in cells in which trafficking has been grossly perturbed by manipulation, particularly overexpression, of any relevant component.

## Methods

### Expression Constructs

The full-length eGFP-wild-type myosin-Vb, eGFP-myosin-Vb tail, mRFP-Rab4a, mRFP-Rab5a, mRFP-Rab8a and mRFP-Rab11a expression constructs were gifts from Jim Goldenring and are based on the peGFP-C2 expression vector (Clontech). The sensitized Y119G mutant eGFP myosin-Vb was created by shuttling a 989-bp *Cla*I/*Bst*EII fragment from pEcho/pcDNA3.1 Y119G myosin-Vb [20]. The control eGFP-tagged myosin-Vb 1IQ expression vector was created by amplifying the eGFP-tagged, wild-type myosin-Vb sequence for the head domain through the first IQ domain and cloning into pYY8.

### Cells and transfection

HeLa cells were cultured as described [20]. For transfections, 1 μg of Lipofectamine 2000 (Invitrogen) was added to 40 μl of OptiMEM (Invitrogen), then mixed with 0.5 μg of DNA diluted into 40 μl of OptiMEM according to manufacturer's instructions. HeLa cells were trypsinized, collected and resuspended in complete medium at a concentration of 1 × 10^6 ^cells/ml. The cell suspension (80 μl) was added to the DNA/liposome mixture and plated as 40-μl dots in live-cell chambers (Bioptechs, Butler, PA) or glass coverslips, incubated for 1–2 h and then flooded with complete medium. Cells were used in experiments 24–48 h after transfection.

### Immunofluorescence

EEA1 was detected using a monoclonal antibody (BD Transduction Laboratories 610456). The primary antibody was detected with Alexa-546- or Alexa-647-labeled goat anti-mouse secondary (Invitrogen). For cell outlines, actin was stained with Alexa-647-labeled phallacidin (Invitrogen).

### Transferrin trafficking, microinjection, and latrunculin treatment

Transfected cells were incubated in serum-free medium for 60 min, then exposed to 10 μg/ml Alexa 546-labeled transferrin (Invitrogen) for 1 min, washed 3 times with PBS and incubated in pre-equilibrated complete medium for the duration of live-cell experiments. For concomitant labeling with transferrin before expression of exogenous eGFP-myosin-Vb (Fig. 2D,E,F), fluorescent transferrin (10 μg/ml) was added to the complete medium used to flood the coverslips during transfection as described above. During live-cell experiments, stage positions of individual transfected cells were stored using MetaMorph (Molecular Devices) and a motorized stage (Prior). To inhibit Y119G myosin-Vb, HeLa cells were injected in the nucleus with 10 mM PE-ADP, 100 mM KCl, 8 mM K-phosphate (pH 7.0), 0.05 mg/ml fixable Alexa 647-labeled dextran (Invitrogen), and 10 mM Mg-ATP using a Harvard Apparatus PLI-100 at 8–15 kilopascals; negative control injections contained the same solution lacking PE-ADP. Assuming an injection volume of ~3% cell volume, the final concentration of PE-ADP was ~300 μM. To inhibit actin polymerization, latrunculin A (Molecular Probes) was added to medium at 2.5 μg/ml.

### Imaging and Quantitation

All images were obtained using a Nikon TE2000E equipped with a Q57 12-bit CCD camera (Roper Scientific) controlled by MetaMorph software. Images were obtained through a 60× (1.2 NA) water-immersion lens that was maintained at 37°C using an objective heater (Bioptechs). For time-lapse movies, images were obtained at a rate of 1 frame/sec over a 1-min time course. Fluorescence imaging of the dextran to identify injected cells was performed following the time-lapse imaging. Instantaneous speeds of individual particles were measured by observers (blindly with respect to experimental conditions) using the Track Points package of MetaMorph and the data were exported to Microsoft Excel. Measurements were obtained from 5–10 cells per condition, and numbers of particles are provided in the Figure 3 legend. Since pixel size produced submicron/sec speed measurement errors, speeds were separated into 3 bins: 0–0.15 μm/sec (stationary and actin-based), 0.16–0.70 μm/sec (both actin- and microtubule-based), and 0.71–1.0 μm/sec (microtubule-based).

## Additional files

All videos are of HeLa cells.

## Abbreviations List

EEA1: early endosomal antigen-1; and PE-ADP: ***N***^6^-(2-phenylethyl)-ADP.

## Authors' contributions

DWP performed most of the experiments, performed most of the data analysis, and designed the project. EJA, PRW, and DZC analyzed particle speeds and assisted in experiments as summer research interns. CMS assisted in performing experiments. JAM designed the project with DWP, performed data analysis, and wrote the manuscript.

## Supplementary Material

Additional file 1 Low-level expression of full-length eGFP-myosin-Vb (green) shows rare, dynamic colocalization (circles) of myosin-Vb and transferrin (red); same cell as shown in Figure 1D–F. Frame acquisition rate, 0.5/sec; elapsed seconds are displayed at lower right. Click here for file

Additional file 2 Overexpression of full-length eGFP-myosin-Vb (green) in the presence of transferrin (red; added at the time of transfection) produces enlarged, less-motile peripheral endosomes decorated with myosin-Vb and containing transferrin at 24 h post transfection; same cells as shown in Figure 2D–F. Frame acquisition rate, 0.5/sec; elapsed seconds are displayed at lower right. Click here for file

Additional file 3 Overexpression of full-length eGFP-myosin-Vb produces enlarged, less-motile peripheral endosomes decorated with myosin-Vb; same field as shown in Figure 2J–L. Frame acquisition rate, 0.5/sec; frame display rate, 3/sec. Click here for file

Additional file 4 Overexpression of full-length eGFP-myosin-Vb (not shown) prevents entry of Alexa 546-labeled transferrin (shown) into perinuclear compartments; same field as Additional file 3. Frame acquisition rate, 0.5/sec; frame display rate, 3/sec. Click here for file

Additional file 5 Overlay of Additional file 3 (myosin-Vb, green) and Additional file 4 (transferrin, red). Click here for file

Additional file 6 Rab11a (red) colocalizes with eGFP-myosin-Vb (green) at high myosin-Vb expression levels. Frame acquisition rate, 0.5/sec; frame display rate, 6/sec. Click here for file

Additional file 7 Rab4 (red) does not colocalize with eGFP-myosin-Vb (green) at high myosin-Vb expression levels. Frame acquisition rate, 0.5/sec; frame display rate, 6/sec. Click here for file

Additional file 8 Rab5 (red) does not colocalize with eGFP-myosin-Vb (green) at high myosin-Vb expression levels. Frame acquisition rate, 0.5/sec; frame display rate, 6/sec. Click here for file

Additional file 9 Chemical-genetic inhibition of sensitized mutant (Y119G) eGFP-myosin-Vb by PE-ADP microinjection does not prevent movement of transferrin-positive particles. Cells were loaded with fluorescent transferrin (red) 30 min before myosin-Vb was inhibited in the center cell by PE-ADP. Frame acquisition rate, 1/sec; frame display rate, 3/sec. Click here for file

Additional file 10 Chemical-genetic inhibition of sensitized mutant (Y119G) eGFP-myosin-Vb by PE-ADP microinjection (cell on left) halts movement of all myosin-Vb-decorated particles, including those being transported via microtubules; same field as Figure 3A. Uninjected control cell is on the right. Frame acquisition rate, 1/sec; frame display rate, 10/sec. Click here for file

Additional file 11 Same conditions as Additional file 10 without a control uninjected cell. Frame acquisition rate, 1/sec; frame display rate, 10/sec. Click here for file

Additional file 12 Negative control cell expressing wild-type eGFP-myosin-Vb; PE-ADP injection (immediately before imaging) does not halt movement of myosin-Vb-decorated particles. Frame acquisition rate, 1/sec; frame display rate, 10/sec. Click here for file
